# Association between Mutations in Papain-like Protease (PLpro) of SARS-CoV-2 with COVID-19 Clinical Outcomes

**DOI:** 10.3390/pathogens11091008

**Published:** 2022-09-03

**Authors:** Jinlin Tan, Zhilong Wu, Peipei Hu, Lin Gan, Ying Wang, Dingmei Zhang

**Affiliations:** 1 School of Public Health, Sun Yat-sen University, Guangzhou 510080, China; 2 The Fourth People’s Hospital of Foshan City, Foshan 528000, China; 3 Jockey Club School of Public Health and Primary Care, The Chinese University of Hong Kong, Hong Kong SAR, China; 4 Huangpu District Center for Disease Control and Prevention, Guangzhou 510700, China; 5 NMPA Key Laboratory for Quality Monitoring and Evaluation of Vaccines and Biological Products, Guangzhou 510080, China

**Keywords:** SARS-CoV-2, PLpro, COVID-19, mutation, outcome

## Abstract

Papain-like protease (PLpro) is important for the replication and transcription of severe acute respiratory syndrome coronavirus 2 (SARS-CoV-2). This study aimed to reveal the PLpro mutations associated with the clinical outcomes of patients. Due to the importance of the S protein in the pathogenicity of SARS-CoV-2, the mutation of the S protein was also analyzed in this study. After downloading the data from the Global Initiative on Sharing Avian Influenza Data (GISAID) database, samples were divided into two groups on the basis of patient status, namely, recovered and dead groups. This study performed a univariate analysis and further explored the association of mutations with patient outcomes through multivariate logistic regression analysis. A total of 138,492 samples were used for analysis. The patients had a mean age of 43.66 ± 21.56 years, and 51.3% of them were female. Multivariate logistic regression results showed that, compared with men, women had a lower risk of dying from coronavirus disease 2019 (COVID-19) (OR = 0.687, 95%CI: 0.638–0.740). Compared with patients aged 17 years and younger, patients aged 18–64 years (OR = 2.864, 95%CI: 1.982–4.139) and patients over 65 years old (OR = 19.135, 95%CI: 13.280–27.572) had a higher risk of death after infection. Compared with the wild type, P78L (OR = 5.185, 95%CI: 2.763–9.730) and K233Q (OR = 5.154, 95%CI: 1.442–18.416) in PLpro were associated with an increased risk of death. A synergistic interaction existed between age and mutations A146D and P78L. The results of the multivariate logistic regression analysis of the data on vaccinated patients demonstrated that, compared with the wild type, the P78L (OR = 3.376, 95%CI: 2.040–5.585) mutation was associated with an increased risk of death. In conclusion, compared with the wild-type PLpro protein, the P78L and K233Q mutations may increase the risk of death in infected individuals. In addition, a synergistic effect existed between age and P78L and K233Q that increased the risk of death in older patients.

## 1. Introduction

Coronavirus disease 2019 (COVID-19), which is caused by severe acute respiratory syndrome coronavirus 2 (SARS-CoV-2), has triggered a worldwide pandemic and spread to more than 200 countries since it was first reported in 2019. Data from the World Health Organization (WHO) show that as of 31 May 2022 at 5:09 pm Central European Time (CET), 526,558,033 cases and 6,287,117 deaths have been confirmed globally [1]. SARS-CoV-2 has a positive and single-stranded RNA genome that is approximately 30,000 bases in length [2,3] and that contains multiple open reading frames (ORF). ORF1a and ORF1b can encode continuous polypeptides, which can generate 16 non-structural proteins (NSPs) after cleavage [4].

Papain-like protease (PLpro), which contains 319 amino acids at positions 745–1063 of NSP3 of SARS-CoV-2 [5], is responsible for the cleavage of NSP1–NSP3 from polyproteins and is essential for viral replication [6]. It can also modulate host immune functions by binding to human ubiquitin-like protein, interferon-stimulated gene 15 (ISG15), and ubiquitin A-52 (UBA52) [5,7]. This ability has long been one of the popular coronavirus-related research topics. Although the PLpros of SARS-CoV-2 and SARS-CoV share 83% sequence identity, their substrate preferences in the host and deubiquitinating and deISGylating (deISG) activities differ considerably [8,9]. Coronaviruses mutate as they spread, and their mutations can lead to changes in structure or function. For example, the Q233E mutation in PLpro of SARS-CoV resulted in reduced deubiquitinase activity [10]. Considering that PLpro is an important enzyme of SARS-CoV-2, its mutations require further research attention.

Different mutations in SARS-CoV-2 may have different effects on the infectivity, virulence, or immune resistance of the virus. Many previous studies focused on the effects of mutations in the Spike (S) protein. The D614G mutation in the S protein can enhance the replication, transmission, and infectivity of SARS-CoV-2 [11,12]. The V367F mutation in the S protein receptor-binding domain (RBD) can also enhance viral infectivity [13]. A study that included 12,343 SARS-CoV-2 genome sequences found that the frequencies of ORF1ab P4715L (i.e., P323L in NSP12) and S protein 614G variants were positively associated with case fatality [14]. A work identified an N501Y mutation in the Spike protein associated with enhanced SARS-CoV-2 infection and transmission [15]. An investigation in the United States found that the E484K mutation in the S protein was associated with immune escape [16].

Genome-wide surveillance is important for focusing on viral mutations. The Global Initiative on Sharing Avian Influenza Data (GIASID) [17,18] is the world’s largest repository of SARS-CoV-2 sequences. As of April 2022, 10 million SARS-CoV-2 sequences have been accumulated in this database. In this study, by using data from GISAID, we focused on the PLpro of SARS-CoV-2 to identify mutations associated with the clinical outcomes of patients. At the same time, we evaluated the potential co-occurrence of mutations in Spike proteins and NSP12_P323L that are known to affect the risk of death in patients with those in PLpro proposed in this study.

## 2. Results

### 2.1. Sociodemographic Characteristics of Patients

We accessed GISAID (https://www.gisaid.org/) [18] on 18 January 2022 and downloaded patient status metadata uploaded between 1 January 2021 and 31 December 2021. We obtained 166,339 entries after the first filtering, with filters including “Host = Human,” “Complete,” “High Coverage,” “Exclude Low Coverage,” “Patient Status,” and “Complete on Collection Date.” We included entries with defined gender, age, and patient status and containing mutations located in NSP3 (*n* = 138,492) after a second filter.

Of the 138,492 entries uploaded by laboratories in 117 countries on six continents, 54.7% (75,707/138,492) were from Europe and 25.3% (34,989/138,492) were from North America.

Out of the 138,492 patients, 135,479 recovered and 3013 died. A total of 51.3% of the cases in the dataset were female. Patients aged 18–64 years represented the largest proportion (69.7%) of the cases, with an average age of 43.66 ± 21.56 for all cases. Univariate analysis revealed that women had a lower risk of death than men. Patients aged 18–64 years and patients aged 65 years and older had a higher risk of death than patients aged 17 years and younger (Table 1).

### 2.2. Mutations

On average, each sample contained 3.26 mutations in the NSP3 protein, with an average of 3.27 mutations in the “Recovered” group and an average of 2.76 in the “Dead” group.

Univariate analysis showed that compared with wild-type SARS-CoV-2, mutations P78L, K233Q, or K93N in PLpro may increase the risk of death in patients, whereas the mutation E162D may reduce the risk of death. However, mutations identified by previous studies, such as Spike_D614G and NSP12_P323L, that may affect the clinical outcomes of patients were not statistically significant in the univariate analysis performed in this study. Among the fifty-four mutations in the S protein, all of them except for D614G, T716I, V70del, H69del, S982A, D1118H, A570D, V1264L, L5F, A243del, and S98F may be related to the clinical outcomes of patients. The detailed results are shown in Table 2.

### 2.3. Age-Subgroup Univariate Analysis

The univariate analysis of PLpro mutations in different age subgroups yielded inconsistent results. Pairwise comparisons through the Breslow-Day test with adjusted statistical significance levels indicated that only the OR values of the K233Q, K93N, and E162D mutations were homogeneous among the three age groups.

The A146D mutation may be associated with an increased risk of mortality in patients aged 18–64 years. In all age groups, the P78L, K93N, and K233Q mutations may be associated with an increased risk of death. Although the E162D mutation had a low frequency, it may be associated with a reduced risk of death in patients. The results of the detailed age-subgroup univariate analysis are shown in Table 3.

### 2.4. Multivariate Logistic Regression Analysis

Gender, age, mutations with *p* < 0.1 in univariate analysis, and mutations identified by previous studies that may affect the clinical outcomes of patients, such as Spike_D614G and NSP12_P323L, were included in the multiple logistic regression analysis. The detailed results are shown in Table A1.

Given the heterogeneity of the OR values of A146D, P78L, and K233Q among different age groups, we also constructed a logistic regression model that included interaction terms. The difference between the two models was statistically significant (*p* < 0.001), and the inclusion of interaction terms was reasonable.

The results of logistic regression including interaction terms revealed that, with other factors being equal, the risk of death in female patients was 0.687 (95%CI: 0.638–0.740) times that in males. When infected with the wild-type virus, patients aged 18–64 years (OR = 2.864, 95%CI: 1.982–4.139) and those aged 65 years and older (OR = 19.135, 95%CI: 13.280–27.572) had a significantly higher risk of death than patients aged 17 years and younger. The K233Q and P78L mutations may increase the risk of death by 5.154 (95%CI: 1.442–18.416) and 5.185 (95%CI: 2.763–9.730) times, respectively, compared with the wild type. Unexpectedly, Spike_D614G, Spike_E484K, Spike_N501Y, and NSP12_P323L were not statistically significant. The A701V, D950N, E1258D, E156G, G142D, P26S, R346K, T732A, and V1176F mutations in the S protein may increase the risk of death by 2.048 (95%CI: 1.246–3.366), 1.587 (95%CI: 1.247–2.021), 1.718 (95%CI: 1.29–2.288), 5.658 (95%CI: 3.199–10.006), 1.637 (95%CI: 1.452–1.844), 1.772 (95%CI: 1.162–2.703), 2.405 (95%CI: 1.341–4.312), 2.485 (95%CI: 1.616–3.821), and 1.771 (95%CI: 1.194–2.628) times, respectively, compared with the wild type. The D1259H, F157del, L18F, N1074S, Q677H, T19R, T20N, T240I, T478K, and V1104L mutations in the S protein may reduce the risk of death by 0.474 (95%CI: 0.318–0.707), 0.209 (95%CI: 0.088–0.494), 0.375 (95%CI: 0.252–0.560), 0.135 (95%CI: 0.069–0.262), 0.501 (95%CI: 0.334–0.753), 0.385 (95%CI: 0.243–0.612), 0.185 (95%CI: 0.098–0.35), 0.059 (95%CI: 0.019–0.184), 0.644 (95%CI: 0.434–0.955), and 0.435 (95%CI: 0.289–0.656) times, respectively, compared with the wild type. The multiplicative interactions between age and mutations A146D, P78L, and K233Q were not statistically significant. The detailed results are shown in Table 4.

The results of additive interaction demonstrated the existence of synergistic interactions between age and mutations A146D or P78L. The risk of death in patients aged 18–64 years infected with the virus with the A146D mutation was 3.571 times as high as the sum of the risks in patients exposed to only a single risk factor. The risk of death in patients aged ≥ 65 years infected with the A146D mutant virus was 3.388 times higher than that in patients exposed to only a single risk factor combined. The risk of death in patients aged 18–64 years infected with the virus with the P78L mutation was 2.137 times as high as the sum of the risks in patients exposed to only a single risk factor. Patients over the age of 65 infected with the P78L mutant virus had a 2.761-times higher risk of death than those exposed to only a single risk factor combined. The detailed results are provided in Table 5.

### 2.5. Analysis of Vaccinated Patient Data

Of the 138,492 entries, 3569 were reported to have been vaccinated. Among the vaccinated patients, 3456 recovered and 113 died. We performed univariate and multivariate analyses on the data on vaccinated patients with breakthrough infections. The results of the univariate analysis are shown in Table 6.

Variables with *p* < 0.1 in univariate analysis and mutations previously found to be potentially associated with patient clinical outcomes were included in further logistic regression. The multivariate logistic regression results showed that women had a lower risk of death than men (OR = 0.589, 95%CI: 0.389–0.893). The P78L mutation may increase the risk of death by 3.376 (95%CI: 2.040–5.585) times compared with the wild type (Table 7). The latter result is similar to the logistic regression results of the total metadata. In addition, the Spike_D950N mutation may increase the risk of death by 6.123 (95%CI: 1.147–32.677) times compared with the wild type, whereas the P681R and V1264L mutations in the S protein may reduce the risk of death by 0.045 (95%CI: 0.005–0.387) and 0.118 (95%CI: 0.015–0.922) times, respectively, compared with the wild type.

## 3. Discussion

Studies conducted early in the pandemic highlighted similar substitution rates for most genes in SARS-CoV-2. For example, the replacement rate of ORF1ab and Spike is approximately 3.5 × 10^−4^ per site per year [19]. The PLpro coding sequence of interest in this study was located in ORF1ab. Since the start of the pandemic, many mutation sites have been found, and some of them have high mutation frequencies. Considering the possible co-occurrence of other mutations in the Spike protein, which is widely recognized to affect patient outcomes, our study identified several mutations in PLpro that may affect the risk of death in patients.

The results of multivariate logistic regression on 138,492 items showed that, compared with the reference sequence, the K233Q and P78L mutations were associated with an increased risk of death in patients. The mutations identified in this study that may affect the patient’s risk of death have occurred in variants previously considered to be variants of concern (VOC) [20], such as the P78L mutation in the Delta VOC and the K233Q mutation in the Gamma VOC. Available evidence suggests that the Beta, Delta, and Gamma VOCs significantly increase the risk of death in patients compared with wild-type SARS-CoV-2 [21,22,23]. These mutations may explain some of the pathogenicity changes in VOCs. No dual P78L and K233Q mutations in PLpro were detected in VOCs, and even among the 138,492 entries included in this study, the frequency of double mutations was less than 0.1%, and triple mutations of the above mutations were absent.

Whether the mutation of SARS-CoV-2 affects clinical outcomes is an issue of wide concern. A recent study found that the frequencies of the D614G mutation in the S protein and the P323L mutation in NSP12 were positively correlated with patient mortality [14]. In this study, in addition to the mutation located in PLpro, we included other mutations that may be related to the clinical outcomes of patients in the multivariate logistic regression model. The results of this study revealed that Spike_D614G, Spike_E484K, Spike_N501Y, and NSP12_P323L mutations, which have been shown to be associated with outcomes, were not statistically different between the “Recovery” group and the “Death” group, demonstrating that these mutations were balanced in the two groups. Furthermore, the larger sample size of this study (*n* = 138,492) compared to the above studies enhances its representativeness.

The results of additive interaction analysis indicated the existence of a significant synergistic interaction between age grouping and the A146D and P78L mutations. The risk of death in patients exposed to the factors of older age and infection with the mutated virus was higher than the sum of the risks in patients exposed to only a single factor. In 1976, Rothman developed the sufficient-component casual model, which may be used to explain the variation in the effects of viral mutations with patient age. In addition to SARS-CoV-2 mutation and patient age, other unrecognized factors that affect patient outcomes exist. Thus, further research is needed to reveal possible complementary etiologies and prevent death outcomes.

Moreover, given that studies have shown that existing immunity can reduce the risk of death after breakthrough infection in patients [24,25], immunity is one of the important factors affecting the risk of death in patients. Therefore, in this study, we selected items related to patients who reported having been vaccinated against COVID-19 for further analysis to corroborate our previous findings. Similar to the results obtained from the analysis of 138,492 items in this study, the results of multivariate analysis revealed that the P78L mutation may increase the risk of death. These findings also confirmed the reliability of our results.

Although our study focused on the pathogenicity of SARS-CoV-2 and revealed mutations in PLpro that may be associated with the clinical outcomes of patients, their underlying mechanisms were not elucidated. PLpro has been widely accepted to modulate immune responses by affecting ubiquitination in host cells [5,7]. Notably, position 233 of PLpro (position 1795 of replica polyprotein 1ab) has been identified as one of the ubiquitination sites of the SARS-CoV-2 protein [5,26]. Therefore, the K233Q mutation in PLpro may suppress host immune responses by regulating the ubiquitination of important proteins, thereby affecting the clinical outcomes of patients. However, whether the 78th position of PLpro has special biological functions remains unclear. Further exploration of the functional, structural, and biological changes associated with the P78L and K233Q mutations in PLpro would be meaningful to reveal the mechanisms that affect the risk of death in patients.

In addition, the data used in this study did not meet random sampling requirements. Therefore, sampling bias may exist. However, we still found mutations associated with clinical outcomes in the PLpro gene of SARS-CoV-2. Our findings could provide evidence for early responses to mutations that could lead to clinically fatal outcomes.

## 4. Materials and Methods

### 4.1. Data Collection and Filtering

The GISAID [18] database (https://www.gisaid.org/) was accessed on 18 January 2022 by using filter conditions that included “Host = Human,” “Complete,” “High coverage,” “Low coverage excluded,” “With patient status,” and “Collection date complete”, and patient status metadata (*n* = 166,339) collected between 1 January 2021 and 31 December 2021 were downloaded. Data containing mutations located in NSP3 with defined gender, age, and patient status (*n* = 138,492) were obtained after applying a second filter.

### 4.2. Classification of Patient Status

According to the data provided, 138,492 entries were divided into two categories.

Entries that included “Dead,” “Death,” “Deceased,” “Demise,” “Died,” “Exitus,” “Expired,” or “Fatal” in the patient status were classified into the “Death” group.

Entries that included “Admitted,” “Alive,” “Ambulatory,” “Asymptomatic,” “Discharge,” “Home,” “Hospitalized,” “Inpatient,” “Live,” “Mild,” “Outpatient,” “Paucisymtpmatic,” “Recovery,” “Symptomatic,” or any of their combinations in the patient status were classified into the “Recovery” group.

### 4.3. Mutation

Mutations were assessed by using “AA substitutions” from the GISAID database. Mutations with frequencies below 1% were discarded. The official reference sequence used by GISAID is hCoV-19/Wuhan/WIV04/2019 (WIV04) with the accession ID EPI_ISL_402124. Given that PLpro is not one of the individual proteins displayed in the AA substitutions, mutations in PLpro are presented in the GISAID database as mutations at positions 745–1063 of NSP3.

### 4.4. Statistical Analysis

Categorical variables were described as frequencies (percentages). Chi-squared test or Fisher’s exact test were used to compare categorical variables. Given that age differences can lead to significant differences in the risk of death after infection with SARS-CoV-2, age-subgroup univariate analysis was performed to explore whether the effects of mutations differed by age. Variables with *p* < 0.1 in univariate analysis and mutations found in previous studies that may affect the clinical outcomes of patients were further included in multivariate logistic regression to explore the effect of patient gender, age, and mutation on mortality. The R package “epiR” was used to calculate the indices of additive interaction: the relative excess risk (RERI), the attributable proportion (AP), and the synergy index (S). The synergy index was used as a summary measure of additive interaction [27]. In all statistical analyses, *p*-values less than 0.05 were considered statistically significant. IBM SPSS statistics 25.0 software, R Statistical Software 4.0.1, and RStudio 1.4.1717 were utilized for statistical analysis.

## 5. Conclusions

Compared with the wild type, the P78L and K233Q mutations in PLpro increased the risk of death in infected individuals. A synergistic effect existed between age and P78L and A146D. This effect increased the risk of death in older patients.

## Figures and Tables

**Table 1:** Sociodemographic characteristics of cases in patient metadata.

	Total (*n*/%) (*n* = 138,492)	Recovery (*n* = 135,749)	Death (*n* = 3013)	OR (95%CI)	*p*
Gender					
Male	67,449 (48.7)	65,713	1736	Reference	-
Female	71,043 (51.3)	69,766	1277	0.693 (0.644–0.745)	**<0.001**
Age, Years					
≤17	15,448 (11.2)	15,392	56	Reference	-
18–64	96,483 (69.7)	95,279	1204	3.473 (2.655–4.543)	**<0.001**
≥65	26,561 (19.3)	24,808	1753	19.422 (14.874–25.362)	**<0.001**

Note: Chi-squared test was used to compare group differences. *p* < 0.05 was considered statistically significant and is highlighted in bold.

**Table 2:** Univariate analysis of mutation in PLpro and Spike protein.

Variables		Total (*n*/%) (*n* = 138,492)	Recovery (*n* = 135,479)	Death (*n* = 3013)	OR (95%CI)	*p*
PLpro_A146D	No	111,952 (80.8)	109,558	2394	Reference	0.052
	Yes	26,540 (19.2)	25,921	619	1.093 (0.999–1.195)	
PLpro_P78L	No	123,293 (89.0)	120,827	2466	Reference	**<0.001**
	Yes	15,199 (11.0)	14,652	547	1.829 (1.665–2.010)	
PLpro_K233Q	No	132,093 (95.4)	129,412	2681	Reference	**<0.001**
	Yes	6399 (4.6)	6067	332	2.641 (2.350–2.969)	
PLpro_K93N	No	134,235 (96.9)	131,361	2874	Reference	**<0.001**
	Yes	4257 (3.1)	4118	139	1.543 (1.298–1.834)	
PLpro_E162D	No	136,843 (98.8)	133,835	3008	Reference	**<0.001**
	Yes	1649 (1.2)	1644	5	0.135 (0.056–0.326)	
NSP12_P323L	No	1343 (1.0)	1309	34	Reference	0.369
	Yes	137,149 (99.0)	134,170	2979	0.855 (0.607–1.204)	
Spike_D614G	No	141 (0.1)	140	1	Reference	0.365 ^a^
	Yes	138,351 (99.9)	135,339	3012	3.116 (0.436–22.281)	
Spike_N501Y	No	1,000,098 (72.3)	98,246	1852	Reference	**<0.001**
	Yes	38,394 (27.7)	37,233	1161	1.654 (1.536–1.782)	
Spike_E484K	No	125,795 (90.8)	123,345	2450	Reference	**<0.001**
	Yes	12,697 (9.2)	12,134	563	2.336 (2.127–2.565)	
Spike_T478K	No	48,697 (35.2)	47,163	1534	Reference	**<0.001**
	Yes	89,795 (64.8)	88,316	1479	0.515 (0.479–0.553)	
Spike_L452R	No	51,890 (37.5)	50,220	1670	Reference	**<0.001**
	Yes	86,602 (62.5)	85,259	1343	0.474 (0.440–0.509)	
Spike_P681R	No	51,987 (37.5)	50,323	1664	Reference	**<0.001**
	Yes	86,505 (62.5)	85,156	1349	0.479 (0.446–0.515)	
Spike_T19R	No	52,942 (38.2)	51,224	1718	Reference	**<0.001**
	Yes	85,550 (61.8)	84,255	1295	0.458 (0.426–0.492)	
Spike_R158del	No	58,792 (42.5)	56,988	1804	Reference	**<0.001**
	Yes	79,700 (57.5)	78,491	1209	0.487 (0.452–0.524)	
Spike_E156G	No	59,756 (43.1)	57,962	1794	Reference	**<0.001**
	Yes	78,736 (56.9)	77,517	1219	0.508 (0.472–0.547)	
Spike_F157del	No	59,748 (43.1)	57,937	1811	Reference	**<0.001**
	Yes	78,744 (56.9)	77,542	1202	0.496 (0.461–0.534)	
Spike_D950N	No	61,266 (44.2)	59,546	1720	Reference	**<0.001**
	Yes	77,226 (55.8)	75,933	1293	0.590 (0.548–0.634)	
Spike_G142D	No	90,972 (65.7)	8849	2123	Reference	**<0.001**
	Yes	47,520 (34.3)	46,630	890	0.799 (0.738–0.864)	
Spike_T95I	No	103,806 (75.0)	101,303	2503	Reference	**<0.001**
	Yes	34,686 (25.0)	34,176	510	0.604 (0.549–0.665)	
Spike_P681H	No	105,019 (75.8)	102,920	2099	Reference	**<0.001**
	Yes	33,473 (24.2)	32,559	914	1.376 (1.272–1.489)	
Spike_T716I	No	111,748 (80.7)	109,348	2400	Reference	0.146
	Yes	26,744 (19.3)	26,131	613	1.069 (0.977–1.169)	
Spike_V70del	No	111,846 (80.8)	109,448	2398	Reference	0.099
	Yes	26,646 (19.2)	26,031	615	1.078 (0.986–1.179)	
Spike_H69del	No	111,852 (80.8)	109,454	2398	Reference	0.098
	Yes	26,640 (19.2)	26,025	615	1.079 (0.986–1.180)	
Spike_S982A	No	111,869 (80.8)	109,473	2396	Reference	0.077
	Yes	26,623 (19.2)	26,006	617	1.084 (0.991–1.186)	
Spike_Y144del	No	111,910 (80.8)	109,529	2381	Reference	**0.012**
	Yes	26,582 (19.2)	25,950	632	1.120 (1.025–1.224)	
Spike_D1118H	No	111,917 (80.8)	109,519	2398	Reference	0.085
	Yes	26,575 (19.2)	25,960	615	1.082 (0.989–1.183)	
Spike_A570D	No	111,974 (80.9)	109,578	2396	Reference	0.061
	Yes	26,518 (19.1)	25,901	617	1.089 (0.996–1.191)	
Spike_L18F	No	129,976 (93.9)	127,320	2656	Reference	**<0.001**
	Yes	8516 (6.1)	8159	357	2.097 (1.874–2.348)	
Spike_V1176F	No	131,318 (94.8)	128,665	2653	Reference	**<0.001**
	Yes	7174 (5.2)	6814	360	2.562 (2.289–2.868)	
Spike_T1027I	No	131,581 (95.0)	128,914	2667	Reference	**<0.001**
	Yes	6911 (5.0)	6565	346	2.548 (2.271–2.857)	
Spike_P26S	No	131,587 (95.0)	128,924	2663	Reference	**<0.001**
	Yes	6905 (5.0)	6555	350	2.585 (2.306–2.898)	
Spike_H655Y	No	131,659 (95.1)	128,985	2674	Reference	**<0.001**
	Yes	6833 (4.9)	6494	339	2.518 (2.242–2.827)	
Spike_R190S	No	132,033 (95.3)	129,350	2683	Reference	**<0.001**
	Yes	6459 (4.7)	6129	330	2.596 (2.309–2.918)	
Spike_D138Y	No	132,061 (95.4)	129,382	2679	Reference	**<0.001**
	Yes	6431 (4.6)	6097	334	2.646 (2.354–2.973)	
Spike_T20N	No	132,168 (95.4)	129,470	2698	Reference	**<0.001**
	Yes	6324 (4.6)	6009	315	2.516 (2.232–2.835)	
Spike_K417T	No	132,411 (95.6)	129,712	2699	Reference	**<0.001**
	Yes	6081 (4.4)	5767	314	2.617 (2.321–2.950)	
Spike_V1104L	No	132,462 (95.6)	129,490	2972	Reference	**<0.001**
	Yes	6030 (4.4)	5989	41	0.298 (0.219–0.406)	
Spike_V1264L	No	133,277 (96.2)	130,364	2913	Reference	0.193
	Yes	5215 (3.8)	5115	100	0.875 (0.715–1.070)	
Spike_T732A	No	134,207 (96.9)	131,385	2822	Reference	**<0.001**
	Yes	4285 (3.1)	4094	191	2.172 (1.870–2.523)	
Spike_A701V	No	134,528 (97.1)	131,656	2872	Reference	**<0.001**
	Yes	3964 (2.9)	2823	141	1.691 (1.423–2.008)	
Spike_K417N	No	134,564 (97.2)	131,682	2882	Reference	**<0.001**
	Yes	3928 (2.8)	3797	131	1.576 (1.319–1.884)	
Spike_D215G	No	134,829 (97.4)	131,943	2886	Reference	**<0.001**
	Yes	3663 (2.6)	3536	127	1.642 (1.370–1.967)	
Spike_D80A	No	134,924 (97.4)	132,036	2888	Reference	**<0.001**
	Yes	3568 (2.6)	3443	125	1.660 (1.383–1.992)	
Spike_L242del	No	1,349,839 (97.5)	132,091	2892	Reference	**<0.001**
	Yes	3509 (2.5)	3388	121	1.631 (1.356–1.963)	
Spike_L5F	No	134,987 (97.5)	132,056	2931	Reference	0.500
	Yes	3505 (2.5)	3423	82	1.079 (0.864–1.348)	
Spike_A243del	No	134,759 (97.3)	13,1926	2833	Reference	<0.001
	Yes	3733 (2.7)	3553	180	2.359 (2.022–2.753)	
Spike_D1259H	No	135,566 (97.9)	132,582	2984	Reference	**<0.001**
	Yes	2926 (2.1)	2897	29	0.445 (0.308–0.643)	
Spike_Q677H	No	135,684 (98.0)	132,696	2988	Reference	**<0.001**
	Yes	2808 (2.0)	2783	25	0.399 (0.269–0.592)	
Spike_A27S	No	136,338 (98.4)	133,399	2939	Reference	**<0.001**
	Yes	2154 (1.6)	2080	74	1.615 (1.277–2.042)	
Spike_R158S	No	136,344 (98.4)	133,356	2988	Reference	**0.001**
	Yes	2148 (1.6)	2123	25	0.526 (0.354–0.781)	
Spike_T240I	No	136,559 (98.6)	133,549	3010	Reference	**<0.001**
	Yes	1933 (1.4)	1930	3	0.069 (0.022–0.214)	
Spike_E1258D	No	136,587 (98.6)	133,640	2947	Reference	**<0.001**
	Yes	1905 (1.4)	1839	66	1.627 (1.270–2.086)	
Spike_N1074S	No	136,703 (98.7)	133,699	3004	Reference	**<0.001**
	Yes	1789 (1.3)	1780	9	0.225 (0.117–0.434)	
Spike_P251L	No	136,807 (98.8)	133,800	3007	Reference	**<0.001**
	Yes	1685 (1.2)	1679	6	0.159 (0.071–0.355)	
Spike_K97E	No	136,870 (98.8)	133,874	2996	Reference	**0.002**
	Yes	1622 (1.2)	1605	177	0.473 (0.293–0.764)	
Spike_S98F	No	137,019 (98.9)	134,030	2989	Reference	0.149
	Yes	1473 (1.1)	1449	24	0.743 (0.495–1.114)	
Spike_R346K	No	137,050 (99.0)	134,120	2930	Reference	**<0.001**
	Yes	1442 (1.0)	11,359	83	2.796 (2.233–3.500)	
Spike_l244del	No	134,759 (97.3)	131,926	2833	Reference	<0.001
	Yes	3733 (2.7)	3553	180	2.359 (2.022–2.753)	
Spike_Y160F and Spike_V159L ^b^	None or only one	136,697 (98.7)	133,698	2999	Reference	**<0.001**
	both	1795 (1.3)	1781	14	0.350 (0.207–0.594)	

Note: Chi-squared test was used to compare group differences. *p* < 0.05 was considered statistically significant and is highlighted in bold. ^a^ Continuity correction. ^b^ Since the correlation coefficient of Spike_Y160F and Spike_V159L was > 0.999 and was statistically significant (*p* < 0.001), the two variables were combined.

**Table 3:** Result of age-subgroup univariate analysis of mutations.

	Age ≤ 17		Age 18–64		Age ≥ 65	
Variables	OR (95%CI)	*p*	OR (95%CI)	*p*	OR (95%CI)	*p*
PLpro_A146D		0.055		**0.004**		0.234
No	Reference		Reference		Reference	
Yes	0.384 (0.139–1.062)		0.794 (0.678–0.930)		1.071 (0.957–1.198) *^,^**	
PLpro_P78L		**<0.001**		**<0.001**		**<0.001**
No	Reference		Reference		Reference	
Yes	3.611 (2.017–6.463)		2.481 (2.172–2.832)		1.719 (1.487–1.986) *^,^**	
PLpro_K233Q		**<0.001** ^a^		**<0.001**		**<0.001**
No	Reference		Reference		Reference	
Yes	5.098 (2.172–11.966)		2.930 (2.468–3.479)		2.196 (1.856–2.598) **	
PLpro_K93N		0.374 ^a,c^		**<0.001**		**<0.001**
No	Reference		Reference		Reference	
Yes	0.996 (0.995–0.997)		1.765 (1.376–2.264)		1.740 (1.356–2.233)	
PLpro_E162D		>0.999 ^b,c^		**0.001**		**0.003**
No	Reference		Reference		Reference	
Yes	0.996 (0.995–0.997)		0.133 (0.033–0.535)		0.205 (0.065–0.641)	

Note: Chi-squared test or Fisher’s exact test was used to compare group differences. Breslow-Day test was used for the homogeneity test of the odds ratio. *p* < 0.05 was considered statistically significant and is highlighted in bold. ^a^ Continuity correction. ^b^ Fisher’s exact test. ^c^ A single zero cell existed in the 2 × 2 table. * At the 0.05 level, the difference in the odds ratio was statistically significant compared with patients aged ≤ 17 years. ** At the 0.05 level, the difference in the odds ratio was statistically significant compared with patients aged 18 to 64.

**Table 4:** Result of the logistic regression model with interactions.

Predictive Variables		OR (95%CI)	*p*
Intercept		-	**<0.001**
gender	Male	Reference	
	Female	0.687 (0.638–0.740)	**<0.001**
Age, years	≤17	Reference	
	18–64	2.864 (1.982–4.139)	**<0.001**
	≥65	19.135 (13.280–27.572)	**<0.001**
PLpro_A146D	No	Reference	
	Yes	1.296 (0.282–5.967)	0.739
PLpro_P78L	No	Reference	
	Yes	5.185 (2.763–9.730)	**<0.001**
PLpro_K233Q	No	Reference	
	Yes	5.154 (1.442–18.416)	**0.012**
PLpro_K93N	No	Reference	
	Yes	1.225 (0.742–2.024)	0.428
PLpro_E162D	No	Reference	
	Yes	0.305 (0.044–2.113)	0.229
Spike_D614G	No	Reference	
	Yes	3.754 (0.501–28.098)	0.198
Spike_E484K	No	Reference	
	Yes	0.734 (0.512–1.054)	0.094
Spike_N501Y	No	Reference	
	Yes	0.984 (0.605–1.601)	0.949
NSP12_P323L	No	Reference	
	Yes	1.036 (0.7–1.533)	0.859
Spike_A222V	No	Reference	
	Yes	0.867 (0.722–1.042)	0.128
Spike_A243del	No	Reference	
	Yes	1.254 (0.046–34.062)	0.893
Spike_A27S	No	Reference	
	Yes	0.910 (0.640–1.295)	0.602
Spike_A570D	No	Reference	
	Yes	1.882 (0.602–5.881)	0.277
Spike_A701V	No	Reference	
	Yes	2.048 (1.246–3.366)	**0.005**
Spike_D1118H	No	Reference	
	Yes	0.563 (0.239–1.327)	0.189
Spike_D1259H	No	Reference	
	Yes	0.474 (0.318–0.707)	**<0.001**
Spike_D138Y	No	Reference	
	Yes	1.637 (0.965–2.776)	0.068
Spike_D215G	No	Reference	
	Yes	0.576 (0.211–1.567)	0.28
Spike_D80A	No	Reference	
	Yes	1.977 (0.547–7.148)	0.298
Spike_D950N	No	Reference	
	Yes	1.587 (1.247–2.021)	**<0.001**
Spike_E1258D	No	Reference	
	Yes	1.718 (1.29–2.288)	**<0.001**
Spike_E156G	No	Reference	
	Yes	5.658 (3.199–10.006)	**<0.001**
Spike_F157del	No	Reference	
	Yes	0.209 (0.088–0.494)	**<0.001**
Spike_G142D	No	Reference	
	Yes	1.637 (1.452–1.844)	**<0.001**
Spike_H655Y	No	Reference	
	Yes	1.157 (0.605–2.213)	0.660
Spike_H69del	No	Reference	
	Yes	0.983 (0.268–3.609)	0.979
Spike_K417N	No	Reference	
	Yes	1.103 (0.547–2.221)	0.784
Spike_K417T	No	Reference	
	Yes	0.822 (0.479–1.411)	0.477
Spike_K97E	No	Reference	
	Yes	1.791 (0.962–3.335)	0.066
Spike_L18F	No	Reference	
	Yes	0.375 (0.252–0.560)	**<0.001**
Spike_L242del	No	Reference	
	Yes	0.718 (0.322–1.603)	0.419
Spike_L244del	No	Reference	
	Yes	0.774 (0.029–21.004)	0.879
Spike_L452R	No	Reference	
	Yes	1.31 (0.946–1.815)	0.104
Spike_N1074S	No	Reference	
	Yes	0.135 (0.069–0.262)	**<0.001**
Spike_P251L	No	Reference	
	Yes	0.930 (0.157–5.496)	0.936
Spike_P26S	No	Reference	
	Yes	1.772 (1.162–2.703)	**0.008**
Spike_P681H	No	Reference	
	Yes	0.757 (0.55–1.041)	0.086
Spike_P681R	No	Reference	
	Yes	0.785 (0.570–1.080)	0.137
Spike_Q677H	No	Reference	
	Yes	0.501 (0.334–0.753)	**0.001**
Spike_R158del	No	Reference	
	Yes	0.644 (0.314–1.322)	0.231
Spike_R158S	No	Reference	
	Yes	1.125 (0.468–2.704)	0.792
Spike_R190S	No	Reference	
	Yes	1.490 (0.673–3.301)	0.325
Spike_R346K	No	Reference	
	Yes	2.405 (1.341–4.312)	**0.003**
Spike_S982A	No	Reference	
	Yes	0.291 (0.082–1.038)	0.057
Spike_T1027I	No	Reference	
	Yes	1.554 (0.831–2.909)	0.168
Spike_T19R	No	Reference	
	Yes	0.385 (0.243–0.612)	**<0.001**
Spike_T20N	No	Reference	
	Yes	0.185 (0.098–0.350)	**<0.001**
Spike_T240I	No	Reference	
	Yes	0.059 (0.019–0.184)	**<0.001**
Spike_T478K	No	Reference	
	Yes	0.644 (0.434–0.955)	**0.029**
Spike_T732A	No	Reference	
	Yes	2.485 (1.616–3.821)	**<0.001**
Spike_T95I	No	Reference	
	Yes	1.014 0.886–1.162)	0.836
Spike_V1104L	No	Reference	
	Yes	0.435 (0.289–0.656)	**<0.001**
Spike_V1176F	No	Reference	
	Yes	1.771 (1.194–2.628)	**0.005**
Spike_V70del	No	Reference	
	Yes	0.632 (0.171–2.334)	0.491
Spike_Y144del	No	Reference	
	Yes	1.433 (1.007–2.040)	**0.046**
Spike_Y160F and Spike_V159L	None or only one	Reference	
	Yes	0.614 (0.211–1.786)	0.371
Age (18–64) * A146D (Yes)		2.347 (0.815–6.759)	0.114
Age (≥65) * A146D (Yes)		2.558 (0.894–7.319)	0.080
Age (18–64) * P78L (Yes)		0.938 (0.502–1.754)	0.841
Age (≥65) * P78L (Yes)		0.631 (0.337–1.183)	0.151
Age (18–64) * K233Q (Yes)		0.69 (0.28–1.702)	0.421
Age (≥65) * K233Q (Yes)		0.456 (0.185–1.122)	0.087

Note: *p* < 0.05 was considered statistically significant and is highlighted in bold.

**Table 5:** The results of additive interaction metrics.

Age, Years	Mutation	RERI (95%CI)	AP (95%CI)	S (95%CI)
18–64	A146D	5.554 (−3.059–14.167)	0.637 (0.349–0.926)	3.571 (1.450–8.793)
≥65	A146D	44.022 (−27.341–115.386)	0.694 (0.372–1.015)	3.388 (1.168–9.827)
18–64	P78L	6.879 (2.969–10.789)	0.494 (0.298–0.690)	2.137 (1.359–3.360)
≥65	P78L	39.296 (20.426–58.166)	0.628 (0.544–0.711)	2.761 (2.193–3.475)
18–64	K233Q	3.169 (−3.354–9.693)	0.311 (−0.148–0.770)	1.527 (0.715–3.261)
≥65	K233Q	21.631 (−16.626–59.889)	0.482 (0.089–0.874)	1.970 (0.911–4.264)

Note: The 95% confidence intervals for relative excess risk of interaction (RERI) and attributable proportion due to interaction (AP) do not include 0, and the 95% confidence intervals for the synergy index (S) do not include 1, which means that there is an additive interaction. The synergy index was used as a summary measure of additive interaction.

**Table 6:** Results of univariate analysis of vaccinated patients.

Variables		Total(*n*/%) (*n* = 3569)	Recovery (*n* = 3456)	Death (*n* = 113)	OR (95%CI)	*p*
Gender	Male	1809 (50.7)	1738	71	Reference	**0.009**
	Female	1760 (49.3)	1718	42	0.598 (0.406–0.882)	
Age, years	≤17	55 (1.5)	54	1	Reference	
	18–64	2804 (78.6)	2762	42	0.821 (0.111–6.076)	0.569 ^b^
	≥65	710 (19.9)	640	70	5.906 (0.805–43.353)	**0.048**
PLpro_A146D	No	3375 (94.6)	3264	111	Reference	
	Yes	194 (5.4)	192	2	0.306 (0.075–1.249)	0.081
PLpro_P78L	No	2910 (81.5)	2829	81	Reference	
	Yes	659 (18.5)	627	32	1.783 (1.173–2.708)	**0.006**
PLpro_K233Q	No	3410 (95.5)	3302	108	Reference	
	Yes	159 (4.5)	154	5	0.993 (0.399–2.469)	0.987
PLpro_K93N	No	3545 (99.3)	3434	111	Reference	
	Yes	24 (0.7)	22	2	2.812 (0.653–12.108)	0.175 ^b^
PLpro_E162D	No	3525 (98.8)	3412	113	Reference	
	Yes	44 (1.2)	44	0	0.968 (0.962–0.974)	0.439 ^a^
NSP12_P323L	No	54 (1.5)	51	3	Reference	
	Yes	3515 (98.5)	3405	110	0.549 (0.169–1.787)	0.536 ^a^
Spike_D614G	No	2 (0.1)	2	0	Reference	
	Yes	3567 (99.9)	3454	113	1.033 (1.027–1.039)	>0.999 ^b^
Spike_N501Y	No	3086 (86.5)	2998	88	Reference	
	Yes	483 (13.5)	458	25	1.860 (1.180–2.931)	**0.007**
Spike_E484K	No	3275 (91.8)	3186	89	Reference	
	Yes	294 (8.2)	270	24	3.182 (1.994–5.079)	**<0.001**
Spike_T478K	No	616 (17.3)	584	32	Reference	
	Yes	2953 (82.7)	2872	81	0.515 (0.339–0782)	**0.002**
Spike_L452R	No	563 (15.8)	534	29	Reference	
	Yes	3006 (84.2)	2922	84	0.529 (0.344–0.815)	**0.003**
Spike_P681R	No	3218 (90.2)	3126	92	Reference	
	Yes	351 (9.8)	330	21	2.162 (1.328–3.520)	**0.002**
Spike_T19R	No	624 (17.5)	592	32	Reference	
	Yes	2945 (82.5)	2864	81	0.523 (0.344–0.795)	**0.002**
Spike_R158del	No	823 (23.1)	783	40	Reference	
	Yes	2746 (76.9)	2673	73	0.535 (0.361–0.793)	**0.002**
Spike_E156G	No	763 (21.4)	727	36	Reference	
	Yes	2806 (78.6)	2729	77	0.570 (0.380–0.854)	**0.006**
Spike_F157del	No	822 (23.0)	782	40	Reference	
	Yes	2747 (77.0)	2674	73	0.534 (0.360–0.791)	**0.002**
Spike_D950N	No	848 (23.8)	833	15	Reference	
	Yes	2721 (76.2)	2623	98	2.075 (1.198–3.593)	**0.008**
Spike_G142D	No	1870 (52.4)	1800	70	Reference	
	Yes	1699 (47.6)	1656	43	0.668 (0.454–0.982)	**0.039**
Spike_T95I	No	2422 (67.9)	2347	75	Reference	
	Yes	1147 (32.1)	1109	38	1.072 (0.721–1.594)	0.730
Spike_P681H	No	3218 (90.2)	3126	92	Reference	
	Yes	351 (9.8)	330	21	2.162 (1.328–3.520)	**0.002**
Spike_T716I	No	3370 (94.4)	3259	111	Reference	
	Yes	199 (5.6)	197	2	0.298 (0.073–1.216)	0.073
Spike_V70del	No	3386 (94.9)	3276	110	Reference	
	Yes	183 (5.1)	180	3	0.496 (0.156–1.578)	0.226
Spike_H69del	No	3386 (94.9)	3276	110	Reference	
	Yes	183 (5.1)	180	3	0.496 (0.156–1.578)	0.226
Spike_S982A	No	3374 (94.5)	3263	111	Reference	
	Yes	195 (5.5)	193	2	0.305 (0.075–1.243)	0.079
Spike_Y144del	No	3368 (94.4)	3258	110	Reference	
	Yes	201 (5.6)	198	3	0.449 (0.141–1.426)	0.163
Spike_D1118H	No	3373 (94.5)	3262	111	Reference	
	Yes	196 (5.5)	194	2	0.303 (0.074–1.236)	0.078
Spike_A570D	No	3373 (94.5)	3262	111	Reference	
	Yes	196 (5.5)	194	2	0.303 (0.074–1.236)	0.078
Spike_L18F	No	3386 (94.9)	3279	107	Reference	
	Yes	183 (5.1)	177	6	1.039 (0.450–2.397)	0.929
Spike_V1176F	No	3408 (95.5)	3300	108	Reference	
	Yes	161 (4.5)	156	5	0.979 (0.394–2.435)	0.964
Spike_T1027I	No	3379 (94.7)	3272	107	Reference	
	Yes	190 (5.3)	184	6	0.997 (0.432–2.300)	0.995
Spike_P26S	No	3408 (95.5)	3300	108	Reference	
	Yes	161 (4.5)	156	5	0.979 (0.394–2.435)	0.964
Spike_H655Y	No	3398 (95.2)	3290	108	Reference	
	Yes	171 (4.8)	166	5	0.918 (0.369–2.280)	0.853
Spike_R190S	No	3410 (95.5)	3302	108	Reference	
	Yes	159 (4.5)	154	5	0.993 (0.399–2.469)	0.987
Spike_D138Y	No	3401 (95.3)	3294	107	Reference	
	Yes	168 (4.7)	162	6	1.140 (0.494–2.634)	0.759
Spike_T20N	No	3411 (95.6)	3303	108	Reference	
	Yes	158 (4.4)	153	5	0.999 (0.402–1.486)	0.999
Spike_K417T	No	3413 (95.6)	3305	108	Reference	
	Yes	156 (4.4)	151	5	1.013 (0.407–2.521)	0.977
Spike_V1104L	No	3280 (91.9)	3168	112	Reference	
	Yes	289 (8.1)	288	1	0.098 (0.014–0.706)	**0.004**
Spike_V1264L	No	3301 (92.5)	3189	112	Reference	
	Yes	268 (7.5)	267	1	0.107 (0.015–0.767)	**0.007**
Spike_T732A	No	3563 (99.8)	3450	113	Reference	
	Yes	6 (0.2)	6	0	0.968 (0.963–0.974)	>0.999 ^b^
Spike_A701V	No	3548 (99.4)	3435	113	Reference	
	Yes	21 (0.6)	21	0	0.968 (0.962–0.974)	>0.999 ^b^
Spike_K417N	No	3535 (99.0)	3422	113	Reference	
	Yes	34 (1.0)	34	0	0.968 (0.962–0.974)	>0.999 ^b^
Spike_D215G	No	3554 (99.6)	3441	113	Reference	
	Yes	15 (0.4)	15	0	0.968 (0.962–0.974)	>0.999 ^b^
Spike_D80A	No	3554 (99.6)	3441	113	Reference	
	Yes	15 (0.4)	15	0	0.968 (0.962–0.974)	>0.999 ^b^
Spike_L242del	No	3550 (99.5)	3437	113	Reference	
	Yes	19 (0.5)	19	0	0.968 (0.962–0.974)	>0.999 ^b^
Spike_L5F	No	3513 (98.4)	3400	113	Reference	
	Yes	56 (1.6)	56	0	0.968 (0.962–0.974)	0.327 ^a^
Spike_A243del	No	3512 (98.4)	3401	111	Reference	
	Yes	57 (1.6)	55	2	1.114 (0.268–4.626)	>0.999 ^a^
Spike_D1259H	No	3487 (97.7)	3374	113	Reference	
	Yes	82 (2.3)	82	0	0.968 (0.962–0.973)	0.181 ^a^
Spike_Q677H	No	3534 (99.0)	3422	112	Reference	
	Yes	35 (1.0)	34	1	0.899 (0.122–6.623)	>0.999 ^a^
Spike_A27S	No	3558 (99.7)	3446	112	Reference	
	Yes	11 (0.3)	10	1	3.077 (0.390–24.243)	0.298 ^b^
Spike_R158S	No	3540 (99.2)	3428	112	Reference	
	Yes	29 (0.8)	28	1	1.093 (0.147–8.106)	0.608 ^b^
Spike_T240I	No	3565 (99.9)	3452	113	Reference	
	Yes	4 (0.1)	4	0	0.968 (0.963–0.974)	>0.999 ^b^
Spike_E1258D	No	3412 (95.6)	3299	113	Reference	
	Yes	157 (4.4)	157	0	0.967 (0.961–0.973)	**0.037 ^a^**
Spike_N1074S	No	3422 (95.9)	3309	113	Reference	
	Yes	147 (4.1)	147	0	0.967 (0.961–0.973)	**0.025**
Spike_P251L	No	3525 (98.8)	3412	113	Reference	
	Yes	44 (1.2)	44	0	0.968 (0.962–0.974)	0.227
Spike_K97E	No	3500 (98.1)	3388	112	Reference	
	Yes	69 (1.9)	68	1	0.445 (0.061–3.233)	0.411
Spike_S98F	No	3560 (99.7)	3447	113	Reference	
	Yes	9 (0.3)	9	0	0.968 (00.963–0.974)	>0.999 ^b^
Spike_R346K	No	3460 (96.9)	3365	95	Reference	
	Yes	109 (3.1)	91	18	7.006 (4.062–12.085)	**<0.001**
Spike_l244del	No	3550 (99.5)	3437	113	Reference	
	Yes	19 (0.5)	19	0	0.968 (0.962–0.974)	>0.999 ^b^
Spike_Y160F and Spike_V159L	None or only one	3568 (100.0)	3455	113	Reference	
	both	1 (0.0)	1	0	0.968 (0.963–0.974)	>0.999 ^b^

Note: Chi-squared test or Fisher’s exact test was used to compare group differences. *p* < 0.05 was considered statistically significant and is highlighted in bold. ^a^ Continuity correction. ^b^ Fisher’s exact test.

**Table 7:** Results of logistic regression model for vaccinated patients.

Predictive Variables		OR (95%CI)	*p*
Intercept		-	0.072
Gender	Male	Reference	
	Female	0.589 (0.389–0.893)	**0.013**
Age, years	≤17	Reference	
	18–64	0.642 (0.084–4.907)	0.669
	≥65	5.224 (0.69–39.563)	0.11
PLpro_P78L	No	Reference	
	Yes	3.376 (2.040–5.585)	**<0.001**
Spike_N501Y	No	Reference	
	Yes	0.242 (0.047–1.235)	0.088
Spike_E484K	No	Reference	
	Yes	2.321 (0.427–12.624)	0.330
Spike_T478K	No	Reference	
	Yes	0.98 (0.014–70.552)	0.993
Spike_L452R	No	Reference	
	Yes	1.068 (0.113–10.131)	0.954
Spike_P681R	No	Reference	
	Yes	0.045 (0.005–0.387)	**0.005**
Spike_T19R	No	Reference	
	Yes	3.156 (0.025–400.287)	0.642
Spike_R158del	No	Reference	
	Yes	–	>0.999
Spike_E156G	No	Reference	
	Yes	1.818 (0.408–8.097)	0.433
Spike_F157del	No	Reference	
	Yes	–	>0.999
Spike_D950N	No	Reference	
	Yes	6.123 (1.147–32.677)	**0.034**
Spike_G142D	No	Reference	
	Yes	0.685 (0.419–1.121)	0.132
Spike_P681H	No	Reference	
	Yes	0.438 (0.077–2.482)	0.351
Spike_V1104L	No	Reference	
	Yes	0.143 (0.019–1.084)	0.060
Spike_V1264L	No	Reference	
	Yes	0.118 (0.015–0.922)	**0.042**
Spike_E1258D	No	Reference	
	Yes	0 (0–.)	0.995
Spike_N1074S	No	Reference	
	Yes	0 (0–.)	0.996
Spike_R346K	No	Reference	
	Yes	3.162 (0.285–35.103)	0.349
NSP12_P323L	No	Reference	
	Yes	0.977 (0.087–10.944)	0.985

Note: *p* < 0.05 was considered statistically significant.

## Data Availability

The datasets used and analyzed in this study are available from the corresponding authors upon reasonable request.

## Appendix A

**Table A1:** Results of the logistic regression model without interactions.

Predictive Variables		OR (95%CI)	*p*
Gender	Male	Reference	
	Female	0.688 (0.638–0.741)	**<0.001**
Age, years	≤17	Reference	**<0.001**
	18–64	3.023 (2.310–3.957)	**<0.001**
	≥65	18.285 (13.988–23.902)	**<0.001**
A890D	No	Reference	
	Yes	3.171 (1.040–9.674)	**0.043**
P822L	No	Reference	
	Yes	4.103 (3.399–4.952)	**<0.001**
K977Q	No	Reference	
	Yes	2.689 (1.074–6.733)	**0.035**
K837N	No	Reference	
	Yes	1.218 (0.736–2.013)	0.443
E906D	No	Reference	
	Yes	0.31 (0.044–2.175)	0.239
Spike_D614G	No	Reference	
	Yes	3.755 (0.501–28.114)	0.198
Spike_E484K	No	Reference	
	Yes	0.731 (0.510–1.047)	0.088
Spike_N501Y	No	Reference	
	Yes	0.984 (0.606–1.597)	0.946
NSP12_P323L	No	Reference	
	Yes	1.065 (0.720–1.576)	0.751
Spike_A222V	No	Reference	
	Yes	0.852 (0.709–1.025)	0.090
Spike_A243del	No	Reference	
	Yes	1.398 (0.047–42.055)	0.847
Spike_A27S	No	Reference	
	Yes	0.908 (0.637–1.293)	0.592
Spike_A570D	No	Reference	
	Yes	1.829 (0.589–5.679)	0.297
Spike_A701V	No	Reference	
	Yes	2.001 (1.219–3.285)	**0.006**
Spike_D1118H	No	Reference	
	Yes	0.557 (0.239–1.301)	0.177
Spike_D1259H	No	Reference	
	Yes	0.473 (0.317–0.706)	**<0.001**
Spike_D138Y	No	Reference	
	Yes	1.636 (0.962–2.785)	0.069
Spike_D215G	No	Reference	
	Yes	0.572 (0.210–1.558)	0.275
Spike_D80A	No	Reference	
	Yes	2.076 (0.571–7.553)	0.268
Spike_D950N	No	Reference	
	Yes	1.585 (1.244–2.018)	**<0.001**
Spike_E1258D	No	Reference	
	Yes	1.736 (1.304–2.311)	**<0.001**
Spike_E156G	No	Reference	
	Yes	5.737 (3.248–10.133)	**<0.001**
Spike_F157del	No	Reference	
	Yes	0.207 (0.087–0.490)	**<0.001**
Spike_G142D	No	Reference	
	Yes	1.639 (1.454–1.848)	**<0.001**
Spike_H655Y	No	Reference	
	Yes	1.194 (0.627–2.272)	0.590
Spike_H69del	No	Reference	
	Yes	0.978 (0.274–3.496)	0.973
Spike_K417N	No	Reference	
	Yes	1.073 (0.534–2.158)	0.843
Spike_K417T	No	Reference	
	Yes	0.796 (0.460–1.377)	0.414
Spike_K97E	No	Reference	
	Yes	1.795 (0.965–3.341)	0.065
Spike_L18F	No	Reference	
	Yes	0.377 (0.253–0.563)	**<0.001**
Spike_L242del	No	Reference	
	Yes	0.723 (0.326–1.605)	0.426
Spike_L244del	No	Reference	
	Yes	0.705 (0.023–21.188)	0.840
Spike_L452R	No	Reference	
	Yes	1.313 (0.949–1.816)	0.100
Spike_N1074S	No	Reference	
	Yes	0.131 (0.067–0.255)	**<0.001**
Spike_P251L	No	Reference	
	Yes	0.91 (0.152–5.444)	0.918
Spike_P26S	No	Reference	
	Yes	1.772 (1.160–2.707)	**0.008**
Spike_P681H	No	Reference	
	Yes	0.744 (0.541–1.024)	0.070
Spike_P681R	No	Reference	
	Yes	0.788 (0.572–1.087)	0.147
Spike_Q677H	No	Reference	
	Yes	0.498 (0.332–0.747)	**0.001**
Spike_R158del	No	Reference	
	Yes	0.646 (0.315–1.327)	0.234
Spike_R158S	No	Reference	
	Yes	1.074 (0.448–2.574)	0.874
Spike_R190S	No	Reference	
	Yes	1.558 (0.704–3.448)	0.274
Spike_R346K	No	Reference	
	Yes	2.422 (1.355–4.330)	**0.003**
Spike_S982A	No	Reference	
	Yes	0.303 (0.087–1.062)	0.062
Spike_T1027I	No	Reference	
	Yes	1.605 (0.861–2.991)	0.136
Spike_T19R	No	Reference	
	Yes	0.376 (0.237–0.597)	**<0.001**
Spike_T20N	No	Reference	
	Yes	0.179 (0.093–0.343)	**<0.001**
Spike_T240I	No	Reference	
	Yes	0.061 (0.019–0.189)	**<0.001**
Spike_T478K	No	Reference	
	Yes	0.647 (0.437–0.959)	**0.030**
Spike_T732A	No	Reference	
	Yes	2.497 (1.626–3.833)	**<0.001**
Spike_T95I	No	Reference	
	Yes	1.018 (0.890–1.166)	0.792
Spike_V1104L	No	Reference	
	Yes	0.436 (0.289–0.657)	**<0.001**
Spike_V1176F	No	Reference	
	Yes	1.783 (1.204–2.641)	**0.004**
Spike_V70del	No	Reference	
	Yes	0.649 (0.180–2.334)	0.508
Spike_Y144del	No	Reference	
	Yes	1.439 (1.013–2.043)	**0.042**
Spike_Y160F and Spike_V159L	None or only one	Reference	
	Yes	0.651 (0.224–1.889)	0.430

Note: *p* < 0.05 was considered statistically significant and is highlighted in bold.
